# Effects of environmental enrichment on behaviour, physiology and performance of pigs — A review

**DOI:** 10.5713/ajas.17.0138

**Published:** 2018-12-21

**Authors:** Mbusiseni Vusumuzi Mkwanazi, Cypril Ndumiso Ncobela, Arnold Tapera Kanengoni, Michael Chimonyo

**Affiliations:** 1 Animal and Poultry Science, School of Agricultural, Earth and Environmental Sciences, University of KwaZulu-Natal, Pietermaritzburg 3209, South Africa; 2 Veterinary Services and Research Department, Joburg Zoo, Private Bag X 13, Parkview 2122, South Africa; 3 College of Agriculture and Environmental Sciences, University of South Africa, Florida Campus, Florida 1709, South Africa

**Keywords:** Exploration, Instinctive Behaviours, Intensive Production Systems, Novelty, Pig Welfare

## Abstract

This paper aims to critically analyse and synthesise existing knowledge concerning the use of environmental enrichment and its effect on behavior, physiology and performance of pigs housed in intensive production systems. The objective is also to provide clarity as to what constitutes successful enrichment and recommend when and how enrichment should be used. Environmental enrichment is usually understood as an attempt to improve animal welfare and to a lesser extent, performance. Common enrichment objects used are straw bedding, suspended ropes and wood shavings, toys, rubber tubings, colored plastic keys, table tennis balls, chains and strings. These substrates need to be chewable, deformable, destructible and ingestible. For enrichment to be successful four goals are essential. Firstly, enrichment should increase the number and range of normal behaviors; secondly, it should prevent the phenomenon of anomalous behaviors or reduce their frequency; thirdly, it should increase positive use of the environment such as space and fourthly it should increase the ability of the animals to deal with behavioral and physiological challenges. The performance, behavior and physiology of pigs in enriched environments is similar or in some cases slightly better when compared with barren environments. In studies where there was no improvement, it should be borne in mind that enriching the environment may not always be practical and yield positive results due to factors such as type of enrichment substrates, duration of provision and type of enrichment used. The review also identifies possible areas that still need further research, especially in understanding the role of enrichment, novelty, breed differences and other enrichment alternatives.

## INTRODUCTION

Intensive indoor-systems are often made up of barren slatted concrete floors with no rooting material [1]. These systems promote boredom and frustration for pigs by precluding the expression of behaviors, which are exhibited, by pigs under normal circumstances [2]. There is a general agreement that pigs raised in these systems are also unable to segregate into natural social groups [3] as they would in their natural habitat. The barren slatted floors may also induce injuries because of behavioral activities such as manipulative oral behaviors, harmful social behaviors and aggressive behaviors directed towards conspecifics [4]. Pigs in barren environments also show physiological signs of stress and poor welfare [5]. The barren slatted floors are slippery especially when the pigs have urinated and defecated in the pens. This, in itself, could lead to undesired injuries during aggressive behavioral activities. Pigs in barren, uninsulated concrete floor environments, are also likely to catch pneumonia during cold seasons especially when the room temperature is not controlled [6].

Animal welfare issues have become an increasing concern to the public as well as the pig industry [7]. The discordance between behavioral needs of pigs and the life afforded to those raised in the intensive indoor systems has created many welfare issues. Pig welfare problems are exacerbated by monotonous and impoverished housing conditions that inhibit the ethological needs of pigs. Nonetheless, intensive indoor systems have a number of advantages such as protection against predators, climate control, control of animals and ease of cleaning and management [8]. These systems, however, offer low levels of environmental stimulation, lack of opportunity for pigs to express their inherent behaviours such as rooting, wallowing and exploring. Welfare issues associated with the use of intensive indoor systems arise from the mere fact that pigs are intelligent and social animals with a wide range of inquisitive behaviours, which are not met under indoor systems [3]. Pigs reared outdoors spend an ample amount of time exploring the environment and foraging. The outdoor system is also inherently good because it provides a more natural habitat [3]. In that way, pigs are able to circumvent aggressive interactions from other pigs by moving away as they are living in a wide-open area.

Environmental enrichment by provision of objects or substrates capable of satisfying the behavioral needs of pigs, is a major method to combat animal welfare problems. These objects or materials have the ability to reduce the amount of time pigs spend on harmful social behaviors. Environmental enrichment is therefore, becoming indispensable in intensive production systems to improve animal welfare while maintaining high productivity of pigs [9,4,10,11]. Pigs in enriched environments are able to perform more of their species – specific behavioral repertoire and accommodate a larger range of behavioral choices [12]. In addition, enriched environments increase activities like exploration, foraging, play and social interaction; which are primary behaviors in pigs. The effects of enrichment can be examined in different approaches such as behavior, physiology and performance of pigs in enriched environments compared to those in barren environments. These aspects all offer insights on how environmental enrichment improves pig welfare. It is of utmost importance, therefore, to integrate available information on the effect of environmental enrichment on pig welfare and performance.

Integrating such information will assist pig farmers in developing valuable enrichment strategies for pigs, such as provision of substrates or materials that will present a stimulus or route for eliciting natural pig behaviors. This paper, therefore, explores existing knowledge concerning environmental enrichment and its effect on behavior, physiology and performance of pigs housed in intensive production systems. These issues are viewed in relation to how enrichment improves behavior and performance of pigs. The review also provides synthesis on how enrichment should be carried out to achieve the intended goals. The review also identifies possible areas that still need further research to improve pig welfare.

## OVERVIEW OF BEHAVIOR AND PHYSIOLOGY OF PIGS UNDER INTENSIVE AND OUTDOOR PRODUCTION SYSTEMS

Pig production systems are essential from the animal welfare viewpoint. In principle, a well-designed production system should improve the behavior of pigs as well as their physiology [13]. Generally, production systems should minimize stress levels while promoting inherent pig behavior. There is a wide range of research that has compared the behavior and physiology of pigs both in outdoor and intensive production systems [8,14]. Blumetto Velazco et al [15] reported that more than 60% of pigs today are reared outdoors with variable access to pastures in the feed. This has been influenced by discourses on environmental sustainability, social benefits and affordability as small and medium farmers are able to keep pigs without having to worry about cost of production [16].

Mosia et al [17] reported that pigs produced outdoor showed different characteristics in terms of physiology, behavior and performance than those in indoor production system. Blumetto Velazco et al [15] reared pigs in both indoor and outdoor systems and reported that active behavior was greater in outdoor pigs than indoor. The pigs kept outdoors showed more levels of activity in the morning and afternoon than pigs reared indoors. Similarly, Johnson et al [18] reported that outdoor reared piglets spent more time engaging in play activity than piglets reared indoors. According to Daza et al [19] pigs in the indoor production system remained physically inactive most of the time. A probable explanation to these findings could be that in the intensive production systems, pigs are restricted in terms of space allocation and hence cannot roam around freely, although other factors may also be contributing. This could also mean that rearing pigs outdoors exposes them to freedom hence they are able to exhibit behaviors that indoor kept pigs cannot perform. Indoor rearing tends to foment competition for resources between pigs thus increasing the occurrence and duration of negative social interactions.

Tomohiro et al [13] housed piglets in indoor and outdoor systems and found no differences in feeding and resting behaviors as well as social interactions. Interestingly, time spent floor – rooting was, however, longer in piglets kept outdoors than their contemporaries in the intensive system. This could possibly mean that outdoor piglets were able to practice their natural behaviors because environmental limitations were removed. Allowing pigs therefore, to live in an outdoor piggery might provide the means to meet their behavioral demands and necessities. Johnson et al [18] reported that indoor sows spent a greater percentage of time lying down and drinking than sows outdoors. The difference in drinking time between these sows could be that outdoor sows have to walk further to obtain water whereas sows indoors have the drinkers available continuously to snout. Blumetto Velazco et al [15] reported that pigs reared indoor had greater serum cortisol levels than their counterparts in outdoor production. Tomohiro et al [13] reported that salivary cortisol levels of the outdoors piglets were lower than that of the indoor piglets. This could be attributed to high stress levels experienced by pigs in indoor production because of limited space allocation and frequent interactions to abnormal behaviours. Glucose concentration in the blood serum was higher in the pigs kept indoors that outdoor production [15]. Pigs reared outdoor tend to have greater plasma glucose concentration than the indoor pigs; however, the production system did not influence plasma lactate, creatine kinase and cortisol [20].

## IMPROVING PIG PRODUCTIVITY THROUGH ENRICHMENT

### What is environmental enrichment?

Environmental enrichment is a very complicated term and is often used inconsistently by several authors [21,22] and it is often associated with the enhancement of the animal’s physical and social environment [23]. This term has been used to refer to changes that involve adding one or more substrates to an animal’s enclosure without enumerating the intended endpoint of the changes [22]. A different school of thought however suggested that the endpoint of environmental enrichment should be to improve the biological functioning of pigs [21]. When enriching the environment, it should be borne in mind, therefore, that successful enrichment should i) increase the number and range of normal behaviors, ii) prevent the phenomenon of anomalous behaviors or reduce their frequency, iii) increase positive use of the environment such as space, and iv) increase the ability of the animals to deal with behavioral and physiological challenges [24,23].

### Types of enrichment

There are different types of enrichment criteria that have been studied in the literature. These include social enrichment, occupational enrichment, physical enrichment and nutritional enrichment. These are supposed to improve the environment of confined pigs and benefit the facilities by ensuring increase on the species – specific behavior and less manipulation of pen structures [25]. The type of enrichment should be related to the targeted behavior and the effectiveness of enrichment is influenced by the form and type of presentation. A variety of these enrichment methods are detailed in literature. This review will distinguish between these different enrichment methods, whereas previous reviews focused on comparing alternative enriched housing systems, such as systems enriched with straw beds and objects only.

### Characteristics of effective enrichment materials

There are different substrates that can be used for enrichment purposes. These substrates may be chewable, deformable, destructible and ingestible [26]. These include substrates like chopped straw bedding, suspended rope and wood shavings, toys, rubber tubing, colored plastic keys, table tennis balls, chains, and strings [27,28]. The behavior of pigs towards enrichment substrates or materials can reveal whether the objects are effective enough as enrichment properties. Enrichment structures subjected at the pig eye level as opposed to the floor are more favorable in maintaining attractiveness to pigs [29,30]. The plausible reasoning to these findings is that substrates or materials presented on the floor level can quickly get dirty with feces and thus become unattractive to pigs.

Presentation and position of an object, therefore, becomes an important factor when enriching the environment. It was reported that an object stimulated interest when suspended at the pig eye level as opposed to being presented on the floor [29]. Furthermore, substrates such as straw, peat or combining ropes with rubber hoses also served as effective forms of enrichment [31]. However, structures such as chains and used car tyres are not to be recommended for long-term use. These structures quickly lose their novelty factor since habituation to a substrate or material occurs rapidly in pigs, as a result reducing their usefulness in stimulating exploration. One way in which novelty can be maintained is by regularly replacing existing objects with new objects to keep the pigs continually exploring.

More so, pigs are more attracted to open rope ends and torn rope ends because they are easier to tear up further [2]. In most reports, straw or objects suspended at the pig eye level are commonly used, although much preference is given to straw as an effective form of enrichment. Its utilization can, however, be problematic to a certain extent as it can create blockages in the slurry systems. To combat this disadvantage, it is pertinent to ensure that whenever straw is applied it is thoroughly chopped especially in slatted floor systems. However, these findings have not indicated clearly much information on breed preferences of objects or materials; and this opens gaps for future research. Identifying such information will help pig farmers on valuable enrichment materials that can be used for different pig breeds. In addition, there is no clear information on how long an object or structure can keep the pigs exploring. It is therefore, pertinent that future research should further explore these areas.

### Impact of pen enrichment on behavior of pigs

Pig behavior is a fast and positive indicator of an animal’s welfare condition. Behavior is measured through video recordings, visual observations and analyzing pigs that are expressing their natural behavior [32]. The use of video cameras to measure behavior gives unbiased behavioral data, as these eliminate human disturbances during observation. Natural instinctive pig behavior is a repertoire of different behaviors pigs exhibit in environments that allow them to carry out behaviors created in the evolutionary process [33].

A good understanding of the behavior of pigs can help to identify and improve pig welfare by designing systems that enhance positive welfare. However, current pig systems do not allow for such as they are often barren slatted concrete floors [24]. Improving the environment, therefore, with enrichment substrates or materials for play or rooting is one solution. Beattie et al [34] reported that environmental enrichment reduces time spent inactive and time spent involved in harmful social and aggressive behaviour while increasing the time spent in exploratory behaviour (Table 1). Oliveira et al [35] indicated that environmental enrichment with wood shavings and hanging toys increases the interaction of animals, allowing them to exhibit natural behaviours.

### Effect of pen enrichment on aggressive behaviors

Aggression among pigs is a common and normal behavior primarily driven by formation of social or dominance hierarchy. Fighting and conflicts are expected to last for a period of between 24 and 72 hours [36]. Aggressive interactions are performed to determine dominance order, with the dominant pigs ranking above the sub - dominant and submissive pigs [36]. However, high levels of aggression can be a nocuous challenge to pig welfare, partly because pig welfare represents a significant cost to economic efficiency [36]. Aggressive behaviors preferentially occur when the natural behavior of pigs is impeded and they are deeply dissatisfied.

Factors such as uncomfortable environment, poor management, and genetics [37] increase aggressive behavior. In addition, limiting housing systems with no enrichment substrates or materials can further exacerbate this overt behavior. Pigs are rooting and foraging species, and when prevented such opportunities they tend to react aggressively towards their pen mates. Bolhuis et al [5] reported that pigs in barren environments display more aggression than pigs in enriched environments. This is because enrichment reduces the level of aggressive interactions amongst the pigs while increasing time spent on substrates or materials. The use of straw has been shown to be effective in reducing aggressive behavior among pigs that are unfamiliar [26]. In contrast to this notion, Morgan et al [38] reported an increase in aggression when pigs were enriched with straw. Arey and co-workers [9], however, found that straw increased activity among pigs but did not reduce fighting between newly mixed pigs.

The number of fights between pigs was not eliminated by provision of straw but was shown to be affected by unfamiliarity to each other. Blackshaw et al [29] used metal and tethers on the floor as well as suspended at the pig eye level as enrichment substrates and reduced aggression. However, Madsen et al [39] used alfalfa hay from a dispenser and reported increased aggression. Beattie et al [34] reported that pigs in an enriched environment had less aggressive encounters than their counterparts in a barren environment. O’Connell et al [40] reported that environmental enrichment reduced the expression of aggressive behavior. Pigs from an enriched environment fought less with unfamiliar animals than their counterparts in a barren environment.

Possible explanations to these differences could be due to substrates or materials used in these studies as enrichment structures. These differences could also be because some of the objects used as enrichment properties were difficult for pigs to manipulate, as they were not chewable or destructible enough to reduce aggression. If the object fails to meet the desired needs of pigs, research reports that pigs may perceive the environment as barren even though it is enriched.

### Effect of pen enrichment on harmful social behavior

Social interaction is another common behavior that is often expressed by pigs both in the wild and in confined environments on barren slatted concrete floors. The welfare of pigs in the intensive indoor systems is often compromised by harmful social behaviors like ear and tail biting. Tail biting is arguably the most serious form of harmful social behavior [32] because of its damaging nature. Its welfare and economic implications present an ongoing problem for the pig production industry. Occurrence of harmful social behaviors is an indication that the environment does not meet the behavioral needs of pigs.

Tail biting and manipulation of pen mates is reduced when bedding or manipulative substrates are provided. Zonderland et al [41] reported that provision of objects like chains and rubber hoses were ineffective in preventing tail biting. In contrast, Van de Weerd et al [24] used straw bedding and concluded that the development of tail biting can be prevented (Table 2). Furthermore, Beattie et al [34] reported that pigs in enriched environments showed less harmful social behaviors such as persistent nosing and biting of pen mates than pigs from barren environments.

### Effect of pen enrichment on rooting or foraging behavior

Pigs reared in outdoor production systems [32] frequently express rooting or foraging behaviors. Rooting forms an important part of a behavioral repertoire, constituting a rewarding experience and meeting a behavioral need for pigs [42]. A number of factors can stimulate rooting. These factors are ranked as age, sex, genotype and novelty. Rooting or foraging in a natural environment has, however, a potential to increase the effect of environmental damage. Hence, it is imperative that at least two factors are considered when allocating rooting materials. The rooting material should be allocated in a way that allows proper manipulation and fighting for access to the material should be minimal [32].

Denying pigs access to rooting materials, results in them chewing and manipulating available materials such as pen fixtures [43]. Studnitz et al [44] examined gilts with either rooting or no rooting substrates and reported that gilts in a group that were prevented from rooting, displayed a higher level of anomalous behavior. On the other hand, gilts with rooting materials rooted more. This indicates that pigs not provided with rooting materials are able to substitute rooting with other behaviors. However, anomalous behaviors like biting and fighting become more prominent if there are no rootable materials. Haskell et al [45] reported that pigs root more when they find novelty. This could explain why pigs provided with rooting material were found to root more. Rooting is an exploratory behavior of high priority and depriving pigs of such leads to increase in abnormal behaviors. There is also clear evidence that continuous access to new environment increases rooting behavior partly by stimulating inspective exploration.

### Effect of pen enrichment on exploratory behavior

Exploratory behavior is a form of appetite behavior that is orientated by a search for feed or concerned about examination of areas that are unfamiliar to the pig [2]. The exploratory behavior serves different purposes. Exploratory behavior includes; nosing floors, nosing fixtures, scraping floors, manipulating fixtures, and chewing [5]. In addition, despite the differences in types of exploratory behavior, pigs would use the same behavioral elements such as rooting, sniffing and chewing to explore. Pigs explore to obtain feed, find attractive places to sleep or to collect general information on their surroundings. The desire to explore can, however, also be motivated by hunger, boredom and novelty [30]. Hunger is an immediate goal for exploration and is motivated by an acute need. If pigs are hungry, they search for feed until found and consume sufficient amounts. Hunger increases the level of exploratory behaviors and *ad libitum* feeding does not eliminate the motivation to explore.

### Role of novelty in relation to exploratory behavior

Exploratory behavior can also be motivated by novelty [2]. Novelty is the quality or state of being new, unusual and interesting. Novelty is an important property involved in initiating and maintaining exploration [38]. The motive to explore may arise if the pig discovers unfamiliar objects or when the pig searches for novelty (inquisitive exploration). This means that pigs will explore and obtain information in an object as long as they have the audacity to do and no other motivation becomes more pronounced than the motivation to explore. However, once the object loses its novelty factors, then pigs may start engaging in aggressive interactions. Bolhuis et al [5] reported that pigs spent substantial proportion of their time on exploratory behavior. Interestingly, when analysis was done per behavioral element, it showed that pigs in an enriched environment displayed more of nosing, scraping floor and chewing than pigs in barren environment.

Pigs in barren environments, however, showed more inclinations of manipulating fixtures. Beattie et al [34] reported that pigs in barren environments spent more time nosing fixtures than pigs in enriched environments. Pigs in enriched environments and having lower floor space spent most time nosing the ground. Scott and co-workers [46] evaluated exploratory behavior in pigs housed in straw bedding and reported that pigs with straw bed spend at most 20% of their time exploring straw. Pigs that were given a toy displayed minimal exploratory behavior. A study that evaluated space requirements and enrichment [47] reported that pigs in a high stocking density and enriched with bars, rags and tyres performed more of exploratory behavior than pigs in lower density and no enrichment. Pigs in enriched environments spent most of their active time on exploratory activities than their counterparts in barren environments. Presumably, because pigs find exploration of pen fixtures or pen mates less satisfying, they prefer objects that are chewable, rootable and destructible for exploratory activities.

### Effect of pen enrichment on manipulative behaviours

The increase in the amount of manipulative social behaviors to pen mates may be injurious and thus leading to negative implications on pig welfare [48]. Manipulative social behaviors can be ranked from belly nosing, ear biting, tail biting and manipulation of other parts other than the above. Behaviors that are termed manipulating other parts can be mainly nibbling, chewing of pen mate’s belly and paw chewing [5].

Peeters and co-workers [48] reported that pigs provided with enrichment substrates spent 27.5%±1.7% of their most active time manipulating the substrate. Pigs in an enriched environment spent less time in pen manipulation than pigs in barren environment [34,49]. Bolhuis et al [5] grouped pigs and reported that pigs housed in barren environments showed more of manipulative behavior than their counterparts did in enriched environments. Furthermore, De Jong et al [47] noticed that pigs in enriched environments spent less time showing manipulative behaviors like nibbling, nosing and massaging. The reduction in pen mate and pen fixtures manipulation observed could be explained by findings from other researchers that introduction of chewable objects to pigs has a huge potential to decrease abnormal behaviors directed towards conspecifics.

### Effect of pen enrichment on play behaviour

Play behavior is not common. However, research suggests that pigs in enriched environment exhibit more of play behavior than pigs in barren environments [5,50,51]. Bolhuis et al [5] categorized play behavior in terms of pivoting, rolling, shaking objects and gamboling. Kelly et al [51] used deep straw and reported an increase in play behaviors. On the contrary, Bolhuis et al [5] reported a decrease in play behavior of pigs from week 5 to 6. Newberry et al [50] noted an increase in play behavior of pigs from week 2 to 6. Nonetheless, the positive effect of enrichment was evident as pigs in enriched environments showed more of play behavior than pigs in barren environments in both studies.

Diminution of play behavior in pigs kept in barren environments could indicate endangered health or welfare because regardless of the environment pigs should be able to play. Spinka et al [52] reported that altered play behavior reduced social skills and behavioral flexibility thus reducing welfare in the long term. Generally, research consistently demonstrates a positive response of providing enrichment materials, in that the level of unwanted anomalous behaviors directed towards pen mates is reduced. Table 1 gives an overview of commonly used enrichment materials and their effect on different behavioral activities in pigs.

### Influence of pen enrichment on number and severity of injuries

Injury scoring is a measurement of aggression and fighting that occurs among pigs. Injury scoring involves the counting of injuries subjectively present over the whole body of the animal [53]. The number of external injuries or wounds counted at regular intervals is often used in studies as a measure of social unrest [1]. Injury scores are generally lower for pigs housed in enriched environments compared to barren housed pigs. Van de Weerd et al [54] tested the effect of using different enrichment structures (bite rite tail chew device, straw rack, feed dispenser, straw and liquid dispenser) and reported no significant differences between treatments on skin injuries. Scott et al [45] also reported that adding enrichment objects to straw-bedded pens did not affect skin injuries. Peeters et al [48] found no differences in skin lesions between control pigs and straw-bedded pigs. Manciocco et al [55] found no evidence of environmental enrichment on injuries. However, sex differences were observed with male pigs having higher percentage of injuries than female pigs. This may be partially explained by the fact that male pigs are always more aggressive and engaging in fights.

### Impact of enrichment on physiological parameters

#### Effect of pen enrichment on body temperature

Body temperature may be useful as an indicator of chronic stress as stressors induce an increase in body temperature [56]. De Jong et al [56] studied the effects of straw bedding on the physiology of pigs kept in straw bedded and barren environments. Pigs from both environments exhibited similar reactions in body temperature in response to different stressors such as relocation, isolation and restraint. Pigs in barren environments had a higher body temperature than pigs in enriched environments. The differences in baseline body temperature may be explained by differences in circadian rhythms. Caldara et al [57] reported that body temperature from the neck; shoulders and leg were similar in pigs kept between compact floor, wood shavings and coffee husk. The rectal temperature was, however, higher in pigs enriched with coffee husks as compared to wood shavings and compact floor. However, further research still needs to be done to evaluate how environmental enrichment affects circadian rhythm or body temperature.

### Influence of pen enrichment on heart and respiration rates

Heart rate is a relatively new method used to assess stress [58]. Measuring heart rate is effective because it is instant, quantitative and non-invasive measure of the control of autonomic nervous system [59]. If pigs are placed in stressful conditions, stressors either increase or decrease heart rate. Heart rate frequency is measured in response to stressors using external heart rate monitors [60]. Changes that occur in the heart rate can be caused by physical as well as physiological factors. Beattie et al [34] reported that pigs from enriched environments have higher average and maximum heart rate when given objects than pigs in barren environment. De Jong et al [56] however, found that the heart rate of pigs in enriched and barren environments did not differ. Stress changes the balance between the sympathetic and parasympathetic branch of the autonomic nervous system. Stressors may cause a shift in balance between these systems and thus result in psychological stress.

If the heart rate variability is higher, it represents a shift of the autonomic balance towards a parasympathetic prevalence whereas lower heart rate represents a shift towards sympathetic prevalence [61]. Heart rate variability refers to physiological variation in the time interval between heart beats. These changes are revealed an occasion of stress, however, research does not clearly indicate whether they are due to the environment in which pigs are reared. Therefore, more research still needs to be done looking at how the environment instigates these changes in the balance of the autonomic nervous system in pigs. Caldara et al [57] studied the effect of rearing pigs in deep bedding, coffee husks and compact floor and found that the respiratory rate did not differ between treatments. The respiratory rate was, however, above the threshold range considered normal for the species at this stage of rearing.

### Influence of pen enrichment on stress hormone concentrations

Cortisol is the primary hormone secreted when pigs are subjected to stressful conditions. This is a steroid hormone secreted by the adrenal gland. Cortisol in plasma or saliva is widely used across species to indicate stress. Fluctuations in cortisol levels may reflect physiological stress, physical stress and animal response to a particular environment [62]. The use of salivary cortisol has been noted to be more useful than plasma cortisol as a measure of stress [62]. This is due to difficulty in understanding the relationship between the hypothalamic pituitary adrenal axis (HPA) and exposure to stress.

It is known that the HPA axis can become accustomed to stress stimuli thus affecting plasma cortisol levels and thereby reducing its reliability as a measure of stress. Plasma cortisol is also influenced by diurnal rhythm, with the time of the day affecting cortisol levels. Cortisol levels are higher during light hours and lower during dark hours [63]. Furthermore, cortisol can be produced in increased levels, during exciting events such as feeding. This means cortisol response can increase due to excitement rather than stress. This makes cortisol an unreliable measure or indicator of stress. Hillmann et al [64] reported that when assessing animal welfare, it seems more reliable to consider the circadian pattern of cortisol concentration instead of only one value per day. Enrichment studies, however, have shown that pigs reared in barren environments experience more stress than pigs in enriched environments [56,48,1]. Klont et al [65] reported higher increase in cortisol from far Klont et al m to slaughter in pigs from the barren environment.

Pearce and Paterson [66] reported that provision of enrichment substrates to crowded male pigs did not influence cortisol concentration. However, pigs not provided with substrates had a higher cortisol concentration indicating a response to stress. In contrast, pigs in enriched environments had a higher salivary cortisol concentration than pigs in barren environments prior to transport [60,56,66]. Similarly, De Jong et al [56] studied physiological effects of using straw bedding and found that pigs in enriched environments had higher cortisol concentrations than pigs in barren environments. Bakare et al [67] and Pambu Gollah et al [68] reported that corticosteroid hormones bind two types of receptors in the brain called glucocorticoid receptors and mineralocorticoid receptors. These receptors give pigs the ability to respond adaptively to their environment whether barren or enriched depending on the balance between these receptors. This simply implies that a disturbed balance may lead to reduced or enhanced responsiveness to the environment. However, researchers have not clearly outlined the mechanism behind these phenomena. It would therefore, be of interest to further research on how the pig environment causes disturbances of these two receptors.

### Effect of pen enrichment on intermediary blood metabolites in pigs

Intermediary blood metabolites interact with the central nervous system to regulate different behavioral patterns of pigs Bakare et al [67]. Blood metabolites can also give an indication of the animal’s nutritional status [62]. Peeters et al [48] studied the use of straw bedding at different weeks on blood metabolites; and the study reported no enrichment effect on blood metabolites such as glucose, lactose and non-esterified fatty acids. Pigs enriched with straw bedding for six weeks had lower creatine kinase compared to pigs that were not provided straw bedding. There is a large gap of research, which still needs to be conducted in this area, especially understanding the role of environmental enrichment on changes in metabolism and the use of different enrichment substrates to explain whether the environment does affect metabolism.

### Influence of pen enrichment on growth performance

The performance of pigs is crucial as it reflects their response particularly to feed given and their living environment. It is therefore imperative to understand variations in performance of pigs in different rearing environments be it indoor, outdoor, enriched and barren environments. This enables pig producers to manage pigs differently to improve the performance and welfare of their pigs. Performance of pigs housed under barren and enriched environments has been studied by a number of authors [69,34,70,48]. Though the environments used in these studies was of similar design, performance variables varied. For example, Beattie et al [34] observed that pigs in enriched environments had higher growth rates, which was attributed to higher average daily feed intake (ADFI). Furthermore, pigs in an enriched environment had a better feed conversion ratio (FCR) than their counterparts from barren environments.

Caldara et al [57] reared pigs on compact floor and deep bedding with wood shavings and reported no differences in performance in the growth phase. However, in the finishing phase pigs housed in compact floor showed better results of weight gain, feed intake and FCR. Peeters et al [48] reported that ADFI and FCR were the same in all treatments. Both environments affected pig performance similarly. Pigs that were enriched for a period of six weeks had higher ADG (average daily gain) than other treatments. In a study that compared different enrichment substrates, it was reported that pigs housed in straw-bedded pens had a higher ADFI than pigs enriched with liquid dispenser and bite rite tail chew device. Furthermore, pigs housed in straw-bedded pens had a higher daily weight gain than their counterparts [54]. Conversely, Jordan et al [25] reported that straw and hay did not influence growth rates of pigs.

Enriching the environment improves the growth rates of pigs. The predisposition to increase stress levels in barren environments could be the reason why these pigs had lower performance rates than pigs in enriched environments. Barnett et al [70] reported that higher levels of stress adversely affected FCR. Differences in management and potential differences in behavior may have altered the performance of pigs in barren environments. The differences in space allowance used in these studies could also explain the variation in performance of pigs. Pearce and Paterson [66] reported that space allowance has a huge potential to affect the performance of pigs due to changes in behavioral requirements. Provision of environmental enrichment in early life of pigs results in reduced reactivity to unfamiliar stimuli later in life. Oostindjer and co-workers [71] studied the effects of environmental enrichment during farrowing and enriched post weaning environment on performance. Pre-weaned piglets housed in enriched environment consumed more feed than piglets in barren pens. During the first day post weaning, feed intake was not greater for piglets in enriched environment.

Feed intake over the first 48 hours post weaning were, however, affected by pre weaning enrichment. Piglets in enriched environment had increased feed intake than barren housed piglets. Piglets in enriched environment had higher growth rates than counterparts in barren environments. Johnson et al [18] working with pigs in outdoor paddocks, reported that piglets kept outdoors had a broader feeding behavioral repertoire compared to indoor piglets. These differences in performance could be explained by that piglets preferred some objects over others or the interest in some of the objects declined as the age was increasing.

### Pen enrichment has a bearing on carcass and pork quality

Carcass characteristics of pigs reared in barren and enriched environments were shown to differ significantly [34,49]. Pigs in enriched environments have been reported to have heavier carcass weights and greater levels of backfat thickness than their counterparts from barren environments [34]. On the contrary, Warris et al [72] reported a decrease in backfat thickness for animals in an enriched environment housed in outside pens. Peeters et al [48] used straw bedding for different weeks and reported that pigs that had straw for 4 weeks had lower meat percentage than pigs given straw for two weeks. Moreover, during slaughter no differences were observed in carcass weight and backfat thickness among pigs in all treatments.

Kloet et al [66] also reported no effect of enriched and barren environments on carcass weight, meat percentage and backfat thickness. No differences were observed in carcass quality (cold carcass weight and backfat thickness measured at P2) in pigs reared in straw bedded pens or object -enriched partly slatted floors [1]. Hill et al [73] compared different enrichment substrates on carcass characteristics and reported no treatment effects for backfat opposite the first rib. However, pigs in a negative treatment had a lower backfat opposite the last rib compared to other treatments. Pigs in the control treatment and enriched with toys plus human interaction had a lower last rib backfat than pigs in human treatment alone. Backfat thickness measured opposite the last lumbar vertebrae, pigs in a negative treatment were leaner than other treatments. These measures show that pigs in the negative treatment were leaner than other treatments.

Consumers are increasingly becoming concerned about pig rearing conditions and pork quality. This is because when pigs are reared in confinement there are certain environmental factors and animal - human interactions that are at play. It is, therefore, pertinent that rearing conditions are improved to reduce the effect they impose on pork quality. Muchenje et al [74] reported that if pigs are precluded from behaving normally, occurrences of abnormal and aggressive interactions increase. Such problems may influence pig behavior, welfare, physiology and subsequently their pork quality. Meat produced by pigs in enriched environments had lower cooking losses and shear force values than pork from pigs reared in barren environments.

These improvements in meat quality were not associated with differences in pre-slaughter stress because pH values were similar in all treatments [34,54]. There were no significant effects found on other meat quality parameters because of rearing environment [34]. Warris et al [72] found that pigs reared indoors compared to outdoor reared pigs in enriched environment produced paler pork products than counterparts in barren environment. Furthermore, postmortem pH and water holding capacity for pigs reared outdoor was reduced. However, loin muscle for pigs reared outdoor had a lower ultimate pH, higher drip loss and higher shear force values [75]. Differences captured from these results may be explained by that pigs in enriched environment had higher levels of intramuscular fat, which is associated with improved tenderness and water holding capacity in pork [34]. Nutritional factors gained by pigs enriched with straw could be another possible reason pigs in enriched environment produced better meat quality than their counterparts in barren environment. Maw et al [76] reported that straw has a potential to influence eating quality because pigs reared on straw produced bacon with stronger meat flavor than pigs reared without bedding. Hill et al [73] reported that meat quality measured by shear force, calorimeter scoring and human sensory evaluation was not affected by rearing conditions. Measurements of pork quality flavor, tenderness and juiciness were not different between the treatments. Similarly, Peeters et al [48] also found no effect of rearing environments on meat quality parameters. These studies were, however, using different pig genotypes, therefore, it can be concluded that genotype differences may explain why there were no effect on pork quality.

### Concluding remarks and aspects that need further research

When providing enrichment properties, it is imperative to assess the outcomes to ensure that enrichment programmes meet the intended goals. Environmental enrichment programs are measures taken to enable species-specific behaviour, to alleviate boredom and to eliminate anomalous behaviours. Individual housing of social species such as pigs could be very stressful to pigs and could compromise performance and welfare. Environmental enrichment is therefore increasingly becoming indispensable. Pigs are very sensitive and intelligent creatures that need special care and to ensure their physical and behavioural wellbeing. Absorbent bedding material such as straw or wood shavings needs to be added to the pens to afford a clean, comfortable, and dry surface. Substrates such as hanging plastic bottles and mobile and colourful balls need to be changed over time to keep high levels of exploring appetite. Also, substrates such as plastic balls presented on the floor level need to be continually cleaned and disinfected as they can easily get dirty with faeces. Studies are required to determine interaction of bedding and hanging so to know the best possible enrichment for pig wellness.

There is also a need of identifying possible interaction between space allowance and enrichment so that effective enrichment can be used in accordance to space allowance. There is a need to recommended thickness of bedding materials. Enrichment substrate may be non- nutritive, but should be non-toxic. For enrichment initiatives to be effective, it is also vital to investigate breed differences, the role of novelty, influence on pig metabolism, the use of enrichment on pregnant sows and breeding boars, role of different feed ingredients and the utilisation of proteomics and genomics in improving welfare of growing pigs.

### Breed differences, carcass grading and pork quality

For sustainable pig agriculture, the information on breed differences and environmental enrichment need to be investigated due to differences in response to stressors. It would be of interest to differentiate behaviours of slow-growing and fast-growing breeds, including the aspect of group size. The extent to which pen enrichment can improve growth performance of slow-growing indigenous pigs is unknown. It has been suggested that different breeds adapt differently to environmental changes and handling conditions. These differences may also have an impact on the quality of pork. Therefore, further research should explore pork quality of pigs subjected to different environmental enrichments.

### Understanding the role of novelty in an enrichment substrate or material

There is also a need to understand how long a substrate or material can keep pigs exploring. Pigs are so quick to lose interest in an object. Therefore, it is of much interest to determine how long it takes a pig to lose novelty in an object. As a result, if welfare guidelines are to be made available to pig producers, they should provide information on such issues. The time required before the enrichment materials are replaced becomes invaluable.

### Effect of environmental enrichment on metabolism

Metabolite assays have traditionally been used as tools to assess responses to diets and diseases. The influence of environmental enrichment on pig metabolism have, however, been neglected. Metabolism is likely to respond to the behavioural activities that pig’s exhibit. The influence of different enrichment substrates on metabolism also need to be understood.

### Exploring alternative feed ingredients as sources of nutritional enrichment

Nutritional enrichment is a form of presenting varied or novel feed types or changing the method of delivering feed. Pigs are usually enriched with straw, hay, mushroom compost, peat and sawdust. Such items can create problems in the slurry system. When these roughages are not properly stored, they can harbour bacteria, fungi and moulds as a result reducing the welfare of pigs. Prevailing conditions such as drought and increasing temperatures are also likely to reduce production of roughages. Therefore, it is necessary to explore other feed ingredients. Feed ingredients such as agricultural by- products can be used as nutritional enrichment. For example, maize cobs can be hung around the pens or be scattered on the bedding as a form of nutritional enrichment.

### Proteomics

There is evidence that rearing of animals in barren and enriched environment affect the expression of proteins. Rampon et al [77] reported that environmental enrichment caused changes in the expression of genes whose products are involved in neuronal structure, plasticity and neurotransmission. However, studies on environmental enrichment and proteomics have focused on rats and cats [78]. Such information is, however, unavailable in pigs. It would, therefore, be of interest to further understand the role of environmental enrichment on the expression of proteins in pigs.

### Use of enrichment on pregnant sows and breeding boars

The use of environmental enrichment in breeding sows is limited, with most research concentrated on studying pre partum nest building and nesting material [9]. Furthermore, research of enrichment on breeding boars has barely received attention from any scientific literature. Therefore, it would be of utmost interest that research should further explore enrichment on these areas. Such work has a huge potential to assist producers in selecting appropriate enrichment materials for their breeding pigs.

## Figures and Tables

**Table 1:** Effects of different enrichment substrate or materials on behavioral activities

Material	Behaviour	Effect	References
Peat+straw	Aggression	↓	Beattie et al [69]
	Exploration directed towards pen mates	↓	
	Exploration directed towards materials	↑	
Tyres in chains	Aggression	=	Schaefer et al [79]
Rubber toys	Aggression	↓	Schaefer et al [79]
Chain+bars+rages+tyres (Change once a week)	Exploration	↑	Pearce and Paterson [66]
Deep straw bedding	Aggression	↓	Bolhuis et al [5]
	Exploration	↓	
Straw bedding	Play behaviour	↓	Beattie et al [34]
	Manipulation directed pen mates	↓	
	Aggression	↓	
	Exploration directed towards pen mates	↓	
	Harmful social behaviour	↓	
Tethers+metal	Aggression	↓	Blackshaw et al [29]
Mobile+two balls	Aggression	↓	Guy et al [49]
	Exploratory+play	↑	

Source, [2].

In the column at left the enrichment materials are listed, in the second column the behaviour is listed in the following order; behaviour redirected towards pen mates, aggression, harmful social behaviours e.g. tail biting, behaviour redirected towards materials or substrate, play behaviour. In the effect column, arrows indicate if the relevant behaviour has been increased or reduced, while equal signs indicate if the behaviour was unaffected. If the desired effects have been achieved arrows are pointing downwards, while if the desired effects have not been achieved the arrows are pointing upward.

**Table 2:** Carcass characteristics measurements from pigs in different housing environment and using different enrichment types

Environment+enrichment type	Carcass weight (kg/d)	Backfat (mm)	Carcass length	Meat percentage	SEM	References
Barren	73.9	77.8	-	-	0.91	Beattie et al [34]
Enriched	11.9	15.1	-	-	0.57	
Control	-	2.77	83.10	-	-	Hill et al [73]
Toys	-	2.51	83.10	-	-	
Human	-	2.69	83.39	-	-	
Human+Toys	-	2.79	82.93	-	-	
Negative treatment	-	2.34	83.72	-	-	

SEM, standard error of the mean.
